# Recapitulating epithelial tumor microenvironment in vitro using three dimensional tri-culture of human epithelial, endothelial, and mesenchymal cells

**DOI:** 10.1186/s12885-016-2634-1

**Published:** 2016-08-02

**Authors:** Surya P. Lamichhane, Neha Arya, Esther Kohler, Shengnan Xiang, Jon Christensen, V. Prasad Shastri

**Affiliations:** 1 Institute for Macromolecular Chemistry, University of Freiburg, Hermann-Staudinger-Haus Stefan-Meier-Straße 31, 79104 Freiburg, Germany; 2 BIOSS—Centre for Biological Signalling Studies, University of Freiburg, Schänzlestraße 18, 79104 Freiburg, Germany; 3 Helmholtz Virtual Institute on Multifunctional Biomaterials for Medicine, Kantstr. 55, 14513 Teltow, Germany

**Keywords:** Multicellular spheroids, Drug screening, Drug resistance, Oxidative stress, Mesenchymal stem cells

## Abstract

**Background:**

Three-dimensional (3-D) cultures of cancer cells can potentially bridge the gap between 2-D drug screening and in vivo xenografts. The objective of this study was to characterize the cellular and extracellular matrix characteristics of spheroids composed of human lung epithelial cells (epi), pulmonary vascular endothelial (endo) cells, and human marrow-derived mesenchymal stems cells (MSCs).

**Methods:**

Spheroids composed of epi/endo/MSCs, termed herein as synthetic tumor microenvironment mimics (STEMs), were prepared by the hanging drop method. Cellular composition and distribution in the STEMs was characterized using fluorescence microscopy. Induction of reactive oxygen species and upregulation of efflux transporters was quantified using fluorometry and PCR, respectively, and phenotypic markers were qualitatively assessed using immunohistochemistry.

**Results:**

STEMs exhibited three unique characteristics not captured in other spheroid cultures namely, the presence of a spheroid core devoid of epithelial cells and primarily composed of MSCs, a small viable population of endothelial cells hypothesized to be closely associated with MSCs within the hypoxic core, and discrete regions with high expression for vimentin and cytokeratin-18, whose co-expression is co-related with enhanced metastasis. Although cells within STEMs show elevated levels of reactive oxygen species and mRNA for ABC-B1, an efflux transporter associated with drug resistance, they exhibited only modest resistance to paclitaxel and gemcitabine in comparison to 2-D tri-cultures.

**Conclusions:**

The epi/endo/MSC spheroid model described herein offers a promising platform for understanding tumor biology and drug testing in vitro.

**Electronic supplementary material:**

The online version of this article (doi:10.1186/s12885-016-2634-1) contains supplementary material, which is available to authorized users.

## Background

Cancer is a multifactorial and dynamic disease that continues to be a challenge to treat [1]. The development of effective tumor therapeutics significantly depends on reliable in vitro screening systems. The absence of reliable in vitro screening models that could recapitulate key aspects of tumor microenvironment such as drug resistance and phenotypic changes to cells is an impediment to the reliable translation of in vitro findings into in vivo clinical models. This poor in vitro-in vivo correlation is one factor that has an adverse impact on drug development costs, which are currently projected to exceed $1.5 billion for each single drug that gains approval [2]. Therefore, there is a need to develop in vitro models that can more accurately reflect the in vivo environment and in vivo efficacy. Towards this long-term objective, 3-D aggregates of tumor cells, commonly referred to as tumor spheroids, are an attractive alternative to 2-D cell culture [3] as they can reproduce many aspects of the tumor microenvironment including paracrine effects, cell-cell interactions, and extracellular matrix deposition [4–6]. Furthermore, 3-D cell culture can recapitulate many of the environmental factors that induce metabolic and oxidative stress in cells within tumors, such as oxygen and nutritional gradients, hypoxia, and the formation of a necrotic core [3]. 3-D spheroids additionally have the potential to reduce the time and costs associated with translation of laboratory findings into animal models, [7] and are also compatible with the next generation high throughput screening technologies [8, 9].

It is well established that tumors are heterogeneous in both cellularity (epithelial, vascular, immune cells, and fibroblasts) and extracellular matrix (ECM) composition [1]. Multicellular tumor spheroid models currently described in the literature are typically generated using either the primary cells from tumor explants or tumor cell lines, and in some instances are co-cultured with fibroblasts or endothelial cells [3, 10, 11]; therefore placing greater emphasis on the interaction between epithelial cells and stromal cells. In this context a tri culture system composed of human breast cancer epithelial cells, fibroblasts and endothelial cells has been described for high-throughput screening [12]. However, the stroma of a solid tumor has in addition to vascular cells and immune cells, tumor-associated fibroblasts, which are believed to be derived from mesenchymal stem cells (MSCs) [13]. There is also evidence that MSCs may serve as precursor to the stromal cells in epithelial tumors [14]. MSCs, in addition to acting as support cells provide physical cues and soluble cues for angiogenesis [15], are also assumed to have an immunomodulatory role and help in driving an aggressive and drug resistant tumor phenotype [16, 17]. It has been shown that MSCs are actively recruited by tumors to aid in their growth and formation and it has been postulated that MSCs have the capacity to aid in the formation of the cancer niche [18], and their recruitment facilitates metastasis in prostate tumors [19]. For example, the co-injection of MSCs with melanoma cells has been demonstrated to promote allogeneic tumor formation by suppressing the host immune response [20]. These observations prompted us to characterize spheroids derived from lung epithelial adenocarcinoma cells (A549) when co-cultured with human MSCs and human pulmonary microvascular endothelial cells (HPMEC). The rationale to include endothelial cells in the spheroid formation was to test the hypothesis that MSCs additionally might play a role in sustaining endothelial cells in the harsh nutrition depleted environment of tumor cores. To the best of our knowledge this is the first study to characterize multicellular spheroids of epithelial, MSCs, and endothelial cells. This multicellular spheroid system, which we have termed synthetic tumor Microenvironment mimics (STEMs), exhibits many traits of mature tumor environments including a necrotic core devoid of epithelial cells, induction of drug resistance markers, and resistance to chemotherapeutics.

## Methods

### Cell culture experiments

The A549 cell line was provided by the BIOSS toolbox (Centre for Biological Signalling Studies, University of Freiburg) and was genotyped and verified by Labor für DNA Analytik (Freiburg, Germany). A549 was cultured in Dulbecco’s Modified Eagle’s Medium (DMEM) supplemented with 10 % fetal bovine serum (FBS) (Life Technologies, Germany), and 100 U/mL penicillin-streptomycin (PAN Biotech, Germany). The cells were cultured to 70–80 % confluency before being trypsinized for spheroid formation_._ Human pulmonary microvascular endothelial cells (HPMEC) were procured from ScienCell (USA) and cultured in endothelial cell growth medium (ScienCell, USA) supplemented with 5 % FBS and 50 U/mL penicillin-streptomycin. Human marrow-derived mesenchymal stem cells were kindly provided by Dr. Andrea Barbero and were obtained from patients under consent in accordance to the regulations of the institution’s ethical committee (University Hospital Basel; Ref Number of local ethical committee: 78/07). MSCs were sub-cultured in alpha-MEM containing 10 % FBS, 1 % penicillin-streptomycin, and 5 ng/ml fibroblast growth factor-2 (FGF2). HEK293 cells (a cell line derived from Human embryonic kidney cells) were obtained from BIOSS Toolbox (University of Freiburg, Germany), were genotyped and verified by Labor für DNA Analytik (Freiburg, Germany). They were sub-cultured in DMEM with 10 % FBS.

### Transduction of A549 cells and HPMECs

#### Preparation of the viral particles

Lentiviral particles were produced in HEK293 cells. HEK293 cells (1x10^6^ cells/well) were seeded in a 6-well plate and allowed to attach overnight, and then incubated with the lentiviral vector and packing vectors in presence of polyethyleneimine (PEI, MW 25Kda, Sigma, Germany) as the transfection agent. 5 μg of DNA (4:3:1 of transfer vector (GFP: pGIPZ (Openbiosystems, RHS4346), RFP: pTRIPZ RFP ires Not1 (BIOSS Toolbox, University of FReiburg)), packaging coding vector (pCMV-dR8.74, Addgene, Plasmid #22036)) and envelope coding vector (pMD2.G, Addgene, Plasmid #12259) were diluted in 250 μL Opti-MEM (Invitrogen, Germany) and 11.25 μl of PEI was mixed rapidly and incubated for 10 min and then added to the HEK293 cells. After 16 h, the cell medium was replaced with 2 ml of fresh medium. The following day, the culture medium was replaced with complete medium based on the target cells (DMEM or ECM) and 36 h after transfection, the medium containing viral particles was harvested and filtered using a 0.2-μm filter, and then stored at −80° Celsius until further use.

#### Transduction of A549 and HPMEC

A549 or HPMEC were were seeded at a density of 7x10^4^ cells/well in a six well plate, and the cells were allowed to attach overnight, and on the following day the medium was changed and the viral particles encoding for the fluorescent protein of interest were added (RFP transduction of A549: 2 ml of viral particles; GFP transduction of HPMEC: 100 μl of viral particles). This step was repeated the following day to ensure robust transduction. After 24 h, 4 μg/ml of puromycin was added to select the transduced cells, and the dead cells were removed during medium change.

### Preparation of STEMs

**Fig. 1:**
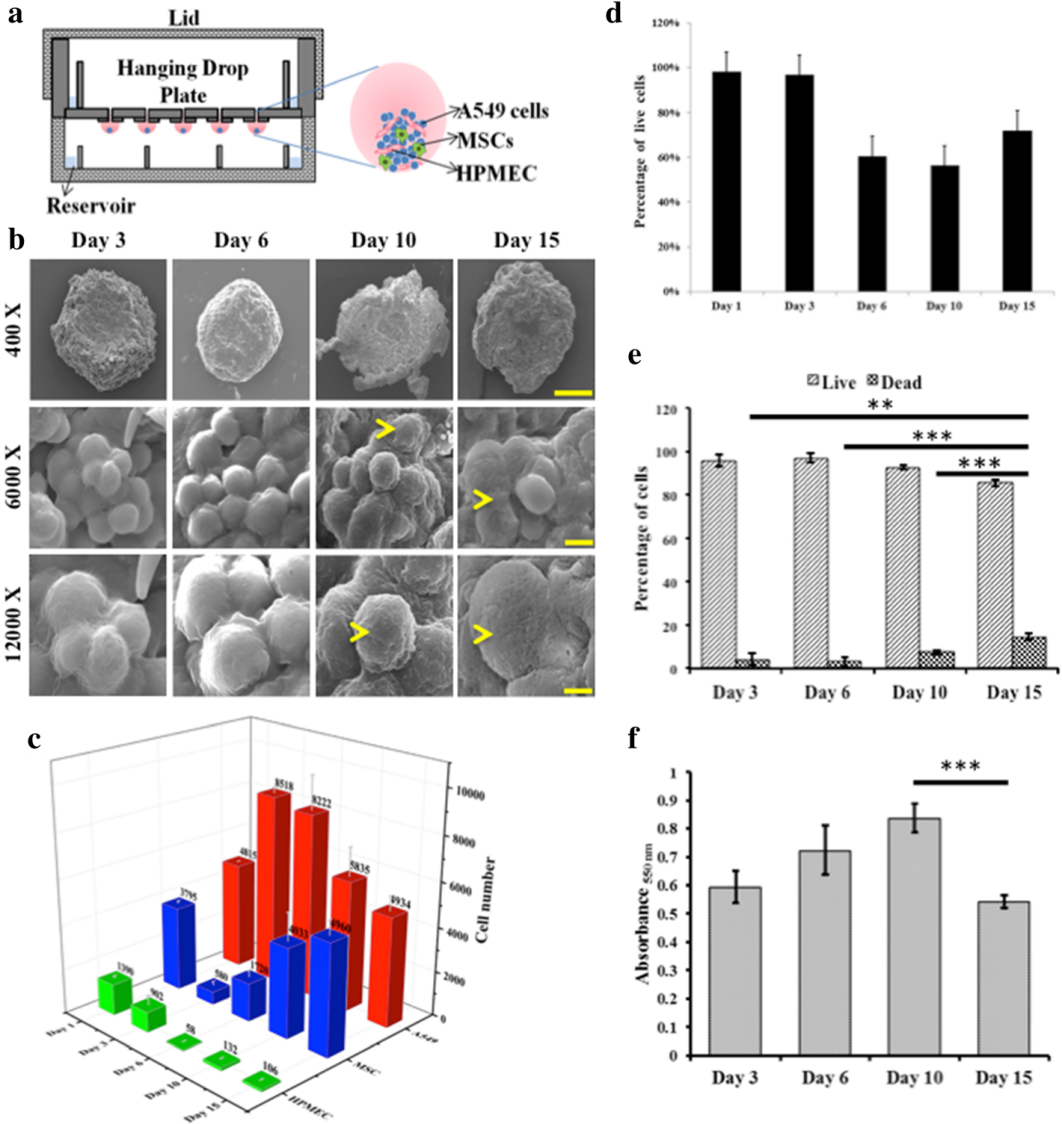
**a** Schematic representation of synthetic tumor microenvironment mimic (STEM) generation using the hanging drop method, **b** Scanning electron micrographs of STEMs at the end of day 3, 6, 10, and 15 (Scale bar 400 X: 200 μm, 6000 X: 10 μm, and 12000 X: 5 μm), **c** Temporal changes to the cellular compositon of the STEMs as determined by FACS (*n* = 3), **d** Fraction of live and dead cells in the STEMs as function of time as assessed by fluorescence activated cell sorting experiment (FACS, *n* = 3), **e** Percentage of live/dead cells as determined by Trypan blue staining (** indicates significance (*p* < 0.01) between day 3 and day 15, and *** indicates sigificance (*p* < 0.001) between day 6 and day 15; and day 10 and day 15). Statistical analysis depicts comparison between the dead populations on each day. **f** Metabolic actitvity (MTT assay) of STEMs as a function (*** indicates (*p* < 0.005) between day 10 and day 15)

Spheroids were prepared using the hanging drop method (Fig. 1a). When the cell cultures reached 70–80 % confluency, cells were harvested by trypsinization, and STEM formation was initiated by combining A549, HPMEC, and MSCs at a ratio of 5:3:2 at a total density of 25×10^3^ cells/25 μl/well in a 96 well hanging drop plate (3-D Biomatrix, USA). The choice of the cell ratio was based on the observation that stromal cells in general comprise a smaller fraction of the tumor, and recent studies have shown that at an A549: MSC ratio of 3:1, MSCs exert a proliferative effect on A549 in vivo [21]. Furthermore, it is has been shown that increased vascularity along the periphery of non-small cell lung carcinoma, of which adenocarcinoma is a subtype, is associated with tumor progression [22]. Therefore, we chose to have a starting cell composition that was high in HPMECs. The wells of the plate were filled with 4 ml of PBS to ensure that there was no evaporation of the cell culture medium from the drop. The medium in the drop was changed every alternate day by removing 2.5 μl of medium from the culture and adding 5 μl of fresh media to the wells.

### Characterization of temporal changes to the cell composition in STEMs

In order to determine the temporal changes in cell population within the STEMs, STEMs were prepared using RFP and GFP expressing A549 and HPMEC respectively, and at pre-determined time points the spheroids were dissociated and the cell population quantified using flow cytometry. Spheroids were collected on day 1, i.e., 24 h after start of the experiment, day 3, 6, 10, and 15, and then transferred into an Eppendorf tube (4 spheroids per tube, *n* =3) and treated with collagenase (0.3 % Sigma Aldrich, Germany) for 30 min, and kept on a shaker maintained at 37 °C. The dissociated cells were resuspended with 300 μl of fluorescence-activated cell sorting (FACS) buffer and stored on ice until the FACS analysis was performed. For each of the experimental conditions, 10,000 viable cells were counted using a Gallios flow cytometer (Beckman Coulter, USA) and the viable cell population was analyzed using Kaluza software (version 1.2, Beckman Coulter) to determine the cellular composition. Percentage of cells that were RFP positive corresponded to A549 population, percentage of cells that were GFP positive corresponded to HPMEC population, and cells that were negative for both GFP and RFP corresponded to the MSC population.

### Fluorescent microscopy of STEMs

STEMs produced using fluorescent protein expressing cells were harvested on day 15 by placing a few drops of PBS through the wells, fixed with 3.7 % formaldehyde and then embedded in OCT (VWR, Germany) overnight. The STEM spheroids were then sectioned into 10 μm sections using a cryo-stat (HYRAX C20, Zeiss), transferred onto slides (Superfrost, VWR, Germany), stained with DAPI nuclear stain, and then imaged using a Zeiss Cell Observer Z1 (Carl Zeiss, Germany) fluorescent microscope. Imaging of spheroids after live/dead staining images were acquired using a Zeiss LSM 510 confocal miscrocope.

### Scanning electron microscopy of STEMs

To investigate the organization of cells within the STEMs as a function of time, spheroids were harvested on day 3, 6, 10, and 15, fixed with 2.5 % glutaraldehyde, dehydrated using graded series of ethanol, and dried in a vacuum desiccator at room temperature for 2 h. The desiccated spheroids were then sputter coated with gold for 60 s before imaging using a scanning electron microscope (SEM) (FEI Quanta 250 FEG). The images were acquired at an accelerating voltage of 20 KV and chamber pressure of 1.14 × 10 Pa at three different magnifications: 400 X, 6000 X, and 12000 X.

### Metabolic acitivty of cells within STEMs

Metabolic activity in STEMs was examined using a 3-(4, 5-dimethylthiazol-2-yl)-2, 5-diphenyltetrazolium bromide (MTT) assay. In the MTT assay, the MTT dye is converted by cellular mitochondrial esterases into an insoluble purple colored formazan that is measured spectrophotometrically and is reflective of metabolic activity of the cell [23]. Spheroids were harvested at day 3, 6, 10, and 15, and incubated with 0.5 mg/ ml of MTT for 3 h . Following this, the MTT solution was aspirated and 100 μl of dimethyl sulfoxide was added to dissolve the purple colored formazan crystals. Absorbance was measured at 550 nm using a Synergy HT microplate reader (Bio-TEK Instruments INC, USA) (*n* = 3).

### Quantification of cell viability within STEMs

The fraction of viable cells within the STEMs was assessed using two quantitative methods: trypan blue exclusion after spheroid dissociation and FACS analysis.

#### Trypan blue exclusion assay

Spheroids were harvested on day 3, 6, 10, and 15, and trypsinized; and the cell suspension was diluted 1:1 with Trypan blue solution (0.4 w/v %), and counted using a hemocytometer (*n* = 3 spheroids with 3 technical repeats). In this assay, live cells exclude the dye and remain unstained while dead cells are stained blue.

#### FACS analysis

The fraction of viable cells within the population of cells that were analyzed was use to determine the fraction of non-viable cells.

### Visualization of live and dead cells within STEMs

Following 15 days of culture, the spheroids were harvested from the hanging drop plate by pipetting 100 μl of PBS through the wells containing spheroids into 1.5 ml microcentrifuge tubes. The spheroids were then washed with PBS and were stained using the live/dead staining kit (Life Technologies, Invitrogen, Germany) by incubating with 6.25 μl each of calcein AM (1:400) and ethidium homodimer (EthD-1) (1:100) at 37 °C for 30 min to visualize live and dead cells and regions of cell death. The spheroids were then imaged with a confocal microscope (Carl Zeiss, Germany) (*n* = 3).

### Oxidative stress assessment in STEMs

Reactive oxygen species (ROS) generation in STEMs was compared to cells grown on 2-D tissue culture polystyrene plates (TCPS). Intracellular ROS was quantified using a fluorescent assay where the non-fluorescent dichlorofluorescein diacetate (DCFH-DA) substrate in the presence of ROS is converted into the fluorescent dichlorofluorescein (DCF). After 15 days of culture, spheroids were transferred to flat-bottomed, dark sided 96 well plates, washed with PBS once, and incubated with 100 μl DCFH-DA for 45 min at 37 °C. Fluorescence was then measured using a plate reader (Bio-TEK, USA) at λ_excitation_ 485 nm and λ_emission_ 535 nm. Finally, the ROS values were normalized with respect to cell number determined by MTT assay (*n* = 7).

### Visualization of hypoxia in STEMs

Spheroids were harvested after 15 days in culture, treated with 200-μM pimonidazole for 3 h , fixed with 3.7 % formaldehyde, and sectioned. Then the sections were permeabilized with 0.1 % Triton-X 100, blocked with 2.5 % goat serum, and incubated with anti-pimonidazole antibody (1:200) (Hypoxyprobe™ Red 549 kit, Hypoxyprobe, Inc., USA) overnight at 4 °C. The samples were then washed with PBS, stained with DAPI, and imaged using a Carl Zeiss microscope, Germany. Since pimonidazole does not bind to necrotic region, the regions of hypoxia can be distinguished from regions of anoxia [24]. The scoring of regions of proliferation and hypoxia was carried out as described by Mikhail et al. [25].

### Immunohistochemistry

The expression of phenotypic markers was analyzed qualitatively using immunohistochemistry. The spheroids and their corresponding 2-D controls were fixed with 3.7 % formaldehyde, and in the case of spheroids, subsequently embedded in OCT before sectioning. The 10 μm thick sections were rehydrated, incubated with 2.5 % goat serum and 0.1 % Triton X-100 in PBS for 1 h. The samples were then incubated with primary antibody CK-18 (1:100, Abcam, Clone: E431-1), fibronectin (1:300, Abcam, Cat No. ab6584), vimentin (1:800, Sigma Aldrich, Germany, Clone V9), and CD 31 (1:100, Abcam, Cat No. ab28364). The sections were then washed with PBS, and incubated with biotinylated secondary antibodies. Color development was performed using the Vectastain Elite kit and diaminobenzidine (DAKO). The samples were then counterstained with hematoxylin and imaged using a Carl Zeiss Z1 Cell observer microscope (Germany) (*n* = 3).

### Expression of ABC-B1 drug resistant marker in STEMs

Gene expression levels of ATP-binding cassette (ABC) sub family B member 1 (*ABC B1*) in STEMs were measured using real time RT-qPCR at the end of day 15. For 2-D samples, the three cell types were cultured together and RNA isolation was performed at 60–70 % confluency using RNAeasy mini kit (QIAGEN), followed by cDNA synthesis by using 250 ng of RNA (Quantitect RT kit, Qiagen). Expression of ABC-B1 was normalized using 18 s rRNA. The sequence of the primers were as follows: ABC-B1: Forward: CAGAGGGGATGGTCAGTGTT; Reverse: CCTGACTCACCACACCAATG; 18srRNA: Forward: CCTGCGGCTTAATTTGACTC; Reverse: AACTAAGAACGGCCATGCAC (*n* = 4).

### Response of STEMs to paclitaxel and gemcitabine

Sensitivity of STEMs at the end of day 15 to escalation in paclitaxel dose (1, 10, 100, and 1000 nM) and gemcitabine (1, 10, 30, and 100 μM) was studied and compared to 2-D triculture at 60–70 % confluency. 48 h after exposure to paclitaxel and gemcitabine, the loss in cell viability was assessed using MTT assay, and the data represented as percentage change with respect to untreated cells (*n* = 3).

### Statistical analysis

All the quantitative data are expressed as mean value ± standard deviation. Statistical analysis was carried out using student’s *t*-test. A *p* value of < 0.05 was considered as statistically significant and * represents *p* < 0.05, ** represents *p* < 0.01, and *** represents *p* < 0.005.

## Results and discussion

### Characterization of STEMs

Since epithelial tumors are heterogeneous with respect to their cell population and also comprise mesenchymal cells, endothelial cell, immune cells, and fibroblasts [26], in this study we aimed to capture this complexity in vitro using a triculture spheroid system derived from A549; human lung epithelial cells, human lung microvascular endothelial cells, HPMEC, and human bone marrow-derived MSCs. Such a system, in theory, could recapitulate some of the in vivo tumor traits, and if so, could provide an interesting platform for tumor staging experiments and drug screening. Although there are a number of techniques available for generation of 3-D cellular aggregates [27], we chose the hanging drop method (Fig. 1a) for generation of STEMs as it is known to yield spheroids of uniform size and promote the formation of tissue-like structures with robust ECM deposition [10]. The spheroids were then characterized for their cellular organization and morphology, composition, cell viability, cell phenotype, stress-related markers, and responsiveness to paclitaxel and gemcitabine.

#### Morphological characterization of STEMs using SEM

As stated earlier the ratio of 5:3:2 of epithelial/endothelial/MSC at the start of the spheroid formation was based on literature observations that adenocarcinomas are highly vascuralized [22] and MSCs when mixed with A549 in the ratio 1:3 exert a proliferative effect [21]. As a first step, a qualitative assessment of the cellular organization in the STEMs was made using SEM at 3, 6, 10, and 15 days after initial spheroid formation (Fig. 1b). The choice of time points was based on the fact that around 15 days reliable measures of tumor microenvironment can be gathered [28] and beyond that period excessive loss of cell viability due to diffusional and nutritional limitations would outweigh the benefits of long-term culture. Furthermore, 15 days was deemed sufficient for the evolution of tumor-related characteristics such as a hypoxic core, phenotypic markers (CK-18, fibronectin) and drug resistance markers (ABC-B1) as discussed later. On Day 3, the cells generally appeared loosely organized. However, on day 6 a consolidation of the cells within the spheroids was evident. Beyond day 6, deposition of ECM was observed (depicted by yellow arrow) and this pattern of cellular organization and ECM deposition is consistent with our recent finding that changes to epithelial tumor volume in vivo initially is primarily due to ECM deposition and not due to an increase in cell numbers [29]. This promoted us to investigate the temporal changes to the cellular composition of the STEMs as discussed below.

#### Temporal changes to cellular composition, viability, and metabolic activity within STEMs

In order to ascertain the changes to the three distinct cell populations within the STEMs as a function of time, HPMEC and A549 were transduced using lentivirus to stably express turbo GFP and turbo RFP, respectively, and the cellular composition of the STEM was quantified by FACS as function of time (Fig. 1c). Based on the cellular population at the initiation of the spheroids of 5:3:2 (A549/HPMEC/MSC), at day 1 it was clear that much of the ECs had perished, and the cellular composition of the STEM was tipped towards A549 and MSC with these two cell populations almost being in equal ratio, and with less than 15 % HPEMCs (A549/HPMEC/MSC; 4.8/1.4/3.8). Interestingly, between days 1 and 3, a 6-fold downward change in MSC population was observed and a further loss of endothelial cells. Considering that the overall fraction of viable cells as per FACS analysis remained relatively unchanged between days 1 and 3 (Fig. 1d) this might allude to a potential proliferation of the A549 population. However, between days 3 and 10, the dead cell population within the spheroids showed a significant increase ranging from 20 to 40 %, as per trypan exclusion analysis of the dissociated STEMs (Fig. 1e), and FACS analysis (Fig. 1d). The cellular composition of the STEMs during this period showed a further significant loss in ECs and the emergence of the MSC fraction with a concurrent downward trend in A549 fraction. Interestingly, MTT metabolic assay also showed higher metabolic activity (Fig. 1f), which may be attributed in part to the plausible proliferation of MSCs. This trend in the changes in the cellular composition and metabolic acitivty continued between days 6 and 10 with an eventual stabilization of the cellular composition within the STEM at day 15. At day 15 the STEMs were composed of an approximately equal fraction of A549 and MSC with an EC fraction of less than 0.1 %. This was also accompanied by a severe reduction in metabolic activity within the STEMs (Fig. 1f). The increase in MSC population with spheroid growth is an important aspect of the STEMs as they mimic the emergence of a stromal population in tumors upon maturation [20]. This upward change in MSC numbers is consistent with literature reports that hypoxia enhances survival [30] and proliferation of MSCs [31], a presumption that is strengthened by the metabolic activity data between days 3 and 6 (Fig. 1f), which shows an increased metabolic activity that coincides generally with the increase in MSC and epithelial cell fraction in the STEMs. Interestingly, the fraction of the ECs during the STEM development between day 6 and 15 remained low and relatively constant at around < 1.0 %; and the significance of this observation is discussed further in the following sections.

### Characterization of cellular microenvironment and cell distribution in STEMs

**Fig. 2:**
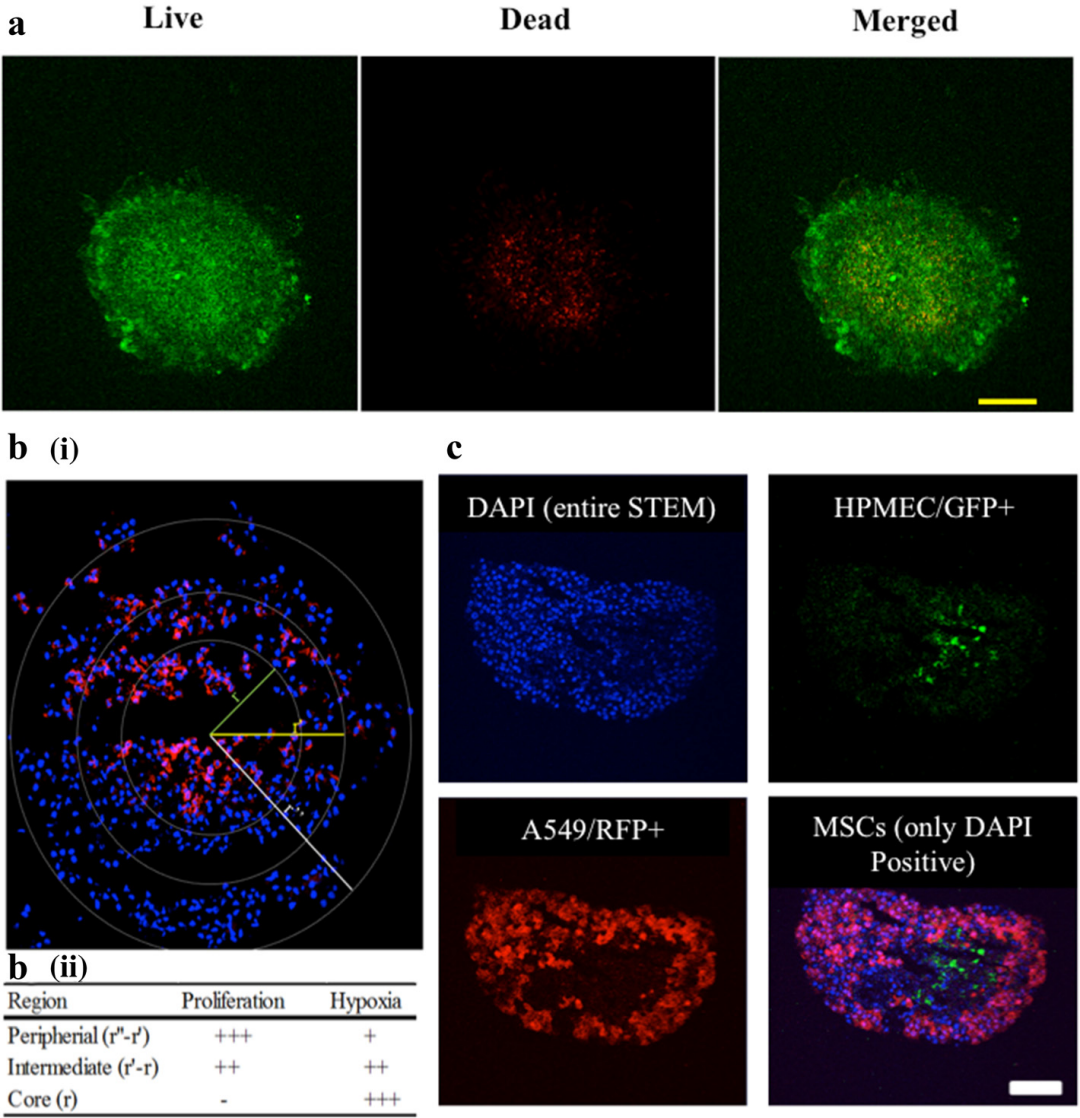
**a** Confocal image of the synthetic tumor microenvironment mimic (STEM) spheroid after staining for live and dead cells (*Green* color represents calcein AM staining indicating live cells, and *red* represents ethidium homodimer staining indicating dead cells) (Scale bar – 200 μm). **b** (i) Immunostaining of STEM at the end of day 15 for hypoxia marker pimonidazole. Hypoxia was confirmed by antibody binding (*pink* color) which is prominent in the interior of the STEM. The nuclei were counter-stained with DAPI. (ii) Scoring of proliferation and hypoxia within various regions of the STEM. The scoring was adapted from Mikhail et al.[25]. **c** Fluorescent micrographs of STEMs generated using turbo GFP expressing human pulmonary microvascular endothelial cells (HPMECs), turbo RFP expressing A549, and MSCs, which turbo GFP and turbo RFP negative cells, i.e. only DAPI positive. Cell nuclei were stained blue using DAPI nuclear stain. DAPI positive, GFP negative and RFP negative cells in the merged image represent MSC populations (represented by *blue* color) (Scale bar 100 μm)

One of the characteristics of a solid tumor is the formation of regions of hypoxia, which in part stems from the presence of aberrant vasculature [1, 3]. Hypoxia in solid tumors is known to promote quiescent cell populations, such as cancer stem cells (CSCs), which alter the responsiveness of tumors to anticancer drugs and radiotherapy [32–34]. Hypoxia is prominent notably in the stromal cell-rich necrotic tumor core, which is believed to foster the survival of a drug resistant tumor cell population that is thought to be responsible for the relapse of a primary tumor [35]. It is well known that spheroids in vitro can also possess a hypoxic core, and the STEMs described herein are no exception. It is evident from the representative image of a spheroid on day 15 shown in Fig. 2a, that the core of the STEM harbors a mixed population of live (green) and dead cells (red), with a higher population of live cells in the periphery. The localization of dead cells within the tumor core may be attributed to restricted nutrient transport into the core as is also seen in vivo [1, 3] and also in other multicellular spheroid systems [36]. The hypoxic nature of the core was confirmed by staining with Hypoxyprobe, a probe for visualization of hypoxia. It was observed that the hypoxic regions were mostly present in the core (r) and intermediate zone (r’-r) of the spheroids and coincide with the populations of dead cells, while cells in the periphery (r΄΄-r΄), frequently referred to as the proliferating zone, showed fewer signs of exposure to hypoxia (Fig. 2b).

#### Characterization of core of STEMs

Generally, when two cell populations are intermixed in 3-D culture, one of the cell population tends to envelope the other and forms a “sphere-in-sphere” organization [37]. However, in a tumor environment, various phenotypically distinct cells coexist in close proximity to one another and often lose some of the organizational restrictions, and exert strong paracrine effects. Using endothelial and epithelial cells expressing eGFP and eRFP, respectively, the organization of the three cell types within the STEMs at day 15 was visualized and qualitatively assessed (Fig. 2c). It is evident that, while the distribution of epithelial and endothelial cells were heterogeneous, MSCs on the other hand were quite homogeneously distributed throughout the spheroid cross-section. However, no “sphere-in-sphere” organization was observed. Remarkably, the core of the spheroid was dominated by MSCs and strikingly devoid of any epithelial cells. However, an even more unexpected finding was that the hypoxic core, in addition to MSCs, harbored a small population of viable endothelial cells as discussed later, which appear closely associated with MSCs. It is well known that MSCs play a crucial role as support cells for endothelial cells [15], and it has been reported that, apoptosis of endothelial cells is inhibited under hypoxic conditions [38]. Therefore, it appears that hypoxia and MSCs might have a synergestic effect on EC survival and such interactions might be promoted in STEMs, however this aspect requires further investigation.

#### Immunohistochemical characterization of STEMs environment for fibrillar fibronectin (FN), and intermediate filament (IF) proteins cytokeratin-18 (CK18) and vimentin

**Fig. 3:**
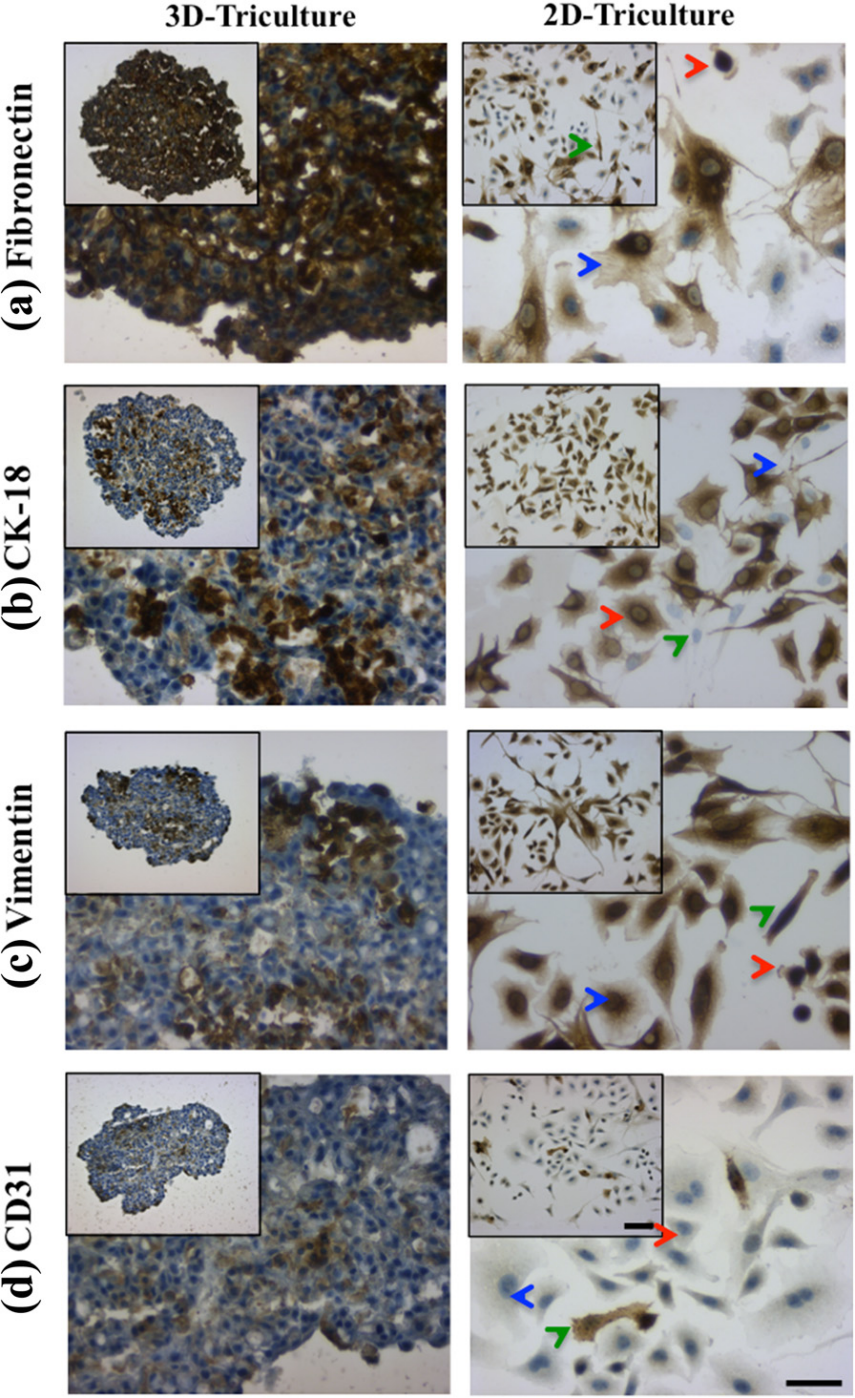
Immunohistochemical staining of STEM cryo-sections at day 15 (*left panel*): (**a**) fibronectin, (**b**) CK-18, (**c**) vimentin, and (**d**) CD-31, and (*right panel*) 2-D tri-culture controls. A549 population indicated by *red arrowhead*, HPMEC population indicated by *green arrowhead*, and MSC population indicated by *blue arrowhead*. Scale bar for low magnification images – 100 μm, scale bar for inset – 50 μm. Negative controls are shown in Additional file 1: Figure S1

FN plays a very important role in organization of cells, cell-cell and cell-matrix interactions [39, 40], and has been implicated in the progression of human lung adenocarcinoma [41]. Dysregulation in FN expression is also thought to contribute to enhanced malignancy [42], suppression of apoptosis [43], and resistance to chemotherapeutics [43, 44]. STEM sections on day 15 showed markedly intense and more uniform staining for FN than observed for cells in triculture in 2-D (Fig. 3a). The negative controls showed no staining, indicating the specificity of the antibody to FN (Additional file 1: Figure S1). This is rather surprising considering the cellular heterogeneity within the STEMs, however such robust expression of FN in 3-D cultures is expected as cells experience more cell-cell contact and paracrine effects in a 3-D environment versus 2-D cultures.

In addition to FN, IF proteins such as CK-18, a marker for epithelial phenotype, and vimentin, an IF protein commonly associated with epithelial–mesenchymal transition (EMT) [1], and also expressed by MSCs, have been shown to be good indicators of epithelial cancer progression [45, 46] and invasiveness [47]. In cervical cancer, an increase in CK18 expression has been associated with disease progression and resistance to cytokine induced apoptosis [48], and in cancers of epithelial origin, upregulation of vimentin is strongly associated with a high degree of tumor growth, invasion, and decreased prognosis [49]. In contrast to FN, staining for both CK 18 and vimentin was both spatially discrete and intense (Fig. 3b and c), and negative controls showed no staining, indicating specificity of the antibodies to the protein of interest (Additional file 1: Figure S1). Since only A549 cells express CK18 (Additional file 2: Figure S2), the formation of discrete foci of intense CK18 (and vimentin expression) staining might be indicative of either epithelial cells demonstrating migratory/invasive phenotype or organizing to form 3-D structures. Although an EMT marker, vimentin is inherently expressed by MSCs. Since MSCs are uniformly distributed within the STEMs at day 15 (Fig. 2c), the appearance of regions with strong expression of vimentin, which we refer to as vimentin “hot spots”, might originate from MSC populations in close association with A549s, or possibly A549s undergoing EMT like transformation. Nevertheless, while this reasoning needs further elucidation, the presence of vimentin “hot spots”, is one of the unique characteristics of the epithelial/endothelial/MSC STEM environment.

#### CD31 staining of STEMs

The presence of endothelial cells in close proximity to MSCs in the core of the STEMs is an intriguing finding that also represents one of the unique characteristics of the epithelial/endothelial/MSC STEM environment. Since it is well established that lung vascular endothelial cells express CD31 [50], staining for CD31 was undertaken in order to ascertain if these eGFP^+^ cells were indeed of endothelial origin. Although, very few CD31^+^ cells were in general observed within the STEMs (Fig. 3d); however, consistent with the observations using labeled cells (Fig. 2c), the primary concentration of CD31^+^ cells was in the vicinity of the hypoxic STEM core. Mislabelling of cell populations could be ruled out as the specificity of CD31 antibody to ECs was verified using a negative control (Additional file 1: Figure S1). Thus, it appears that MSC-endothelial cell interactions are promoted within the STEMs, and more prominently in hypoxic regions. Such interactions are plausible considering that in the past few years compelling evidence have emerged for a crosstalk and interdependency between ECs and MSCs [51] and cancer cells [52]. Furthermore, in angiogenesis, it is well known that pericytes, which are MSC-like cells, act as physical scaffolds to support vascular cells and support angiogenesis [15]. It is also known that mural cells found within vasculature can give rise to multi-lineage MSCs [15]. Since it has also been shown the ECs arising from gliomas undergo the same genomic alteration as the tumor cells [53], taken in sum these observation allude to a more complex interplay between MSCs, tumor cells and ECs. Therefore, our finding that ECs can survive within the oxygen depleted environment of the STEM core is important for two reasons as: (1) it suggests that the hypoxic regions within tumors might, in addition to CSCs, harbor a genetically different sub-population of ECs and (2) if such EC populations are identified in vivo, the STEM platform provides a means to investigate changes to endothelial cell phenotype ex vivo in a controlled setting.

### Characterization of drug resistance markers expressed by STEMs and response of STEMs to paclitaxel and gemcitabine

**Fig. 4:**
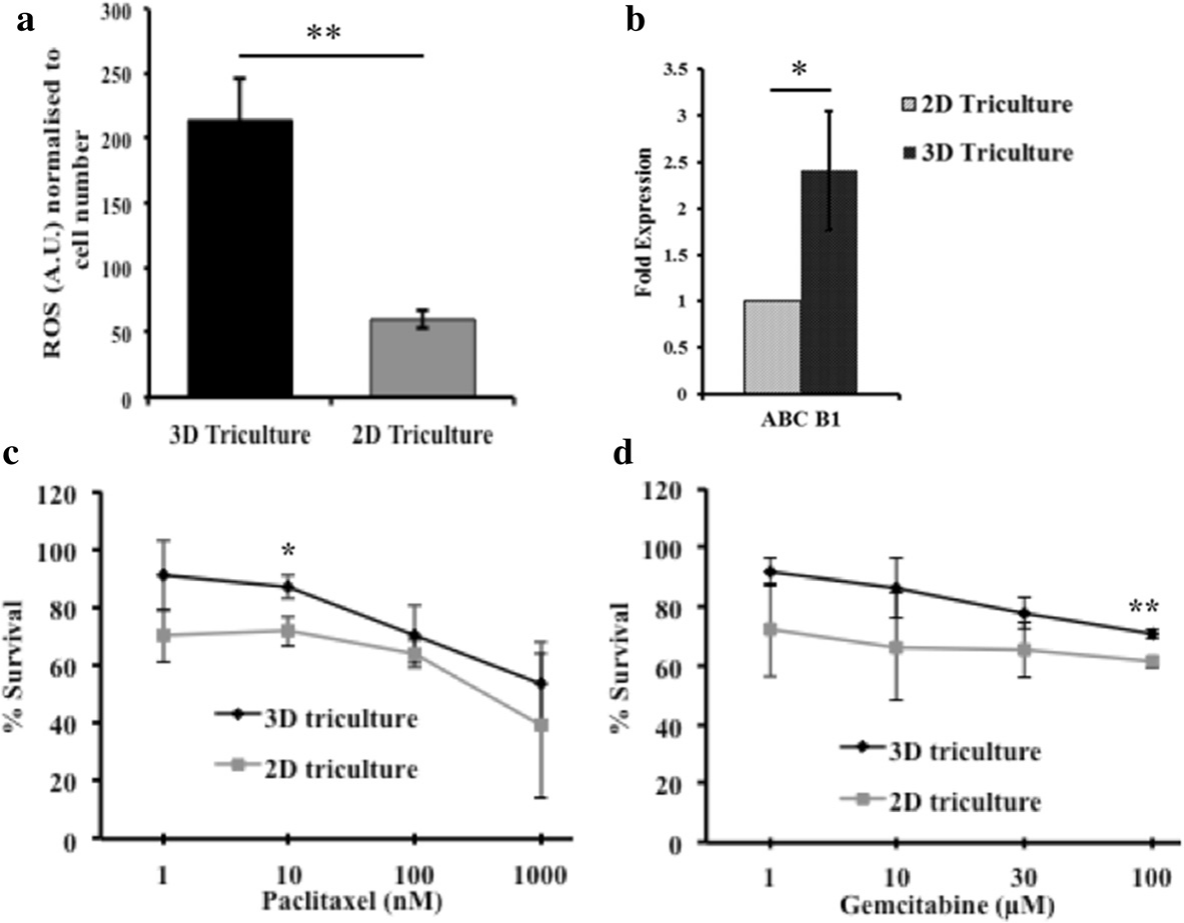
(**a**) Quantification of reactive oxygen species (arbitrary units) induction in synthetic tumor microenvironment mimic (STEM) (at the end of day 15) and 2-D tissue culture polystyrene (TCPS) (at the end of 24 h) normalized to cell number. (** indicates significance (*p* < 0.01) between STEM and 2-D TCPS, *n* = 3). (**b**) ABC-B1 mRNA expression levels in STEM (at the end of day 15), and 2-D TCPS as assessed by real-time PCR. Expressions are normalized to 18 s rRNA and 2-D TCPS (* indicates significance (*p* < 0.05) between 2-D TCPS and STEM) and (**c** & **d**), Cell viability (normalized with respect to untreated control) of STEM and cells grown on 2-D TCPS following exposure to paclitaxel and gemcitabine at the end of 48 h of treatment ((* indicates (*p* < 0.05) between STEM and 2-D TCPS, 10 nM paclitaxel and (*** indicates (*p* < 0.005) between STEM and 2-D TCPS, 100 μm, gemcitabine (*n* = 3))

The presence of a heterogeneous population of cells with varying metabolic needs, when coupled with nutrient diffusion limitations, can promote a stress response in cells. It is known that hypoxia and reactive oxygen species (ROS) production are closely related and can influence several aspects of tumor biology like angiogenesis and pathological alterations in various metabolic pathways [54]. It has been reported that elevated ROS levels in cancer cells can influence their proliferation, survival, resistance to chemotherapy, metastatic potential, and promote stemness [55, 56]. ROS levels in STEM cultures were compared to 2-D tri-cultures and it was observed that the production of ROS in the STEM environment was demonstrably higher (4–5 fold greater) as compared to cells grown on 2-D tissue culture polystyrene (TCPS) (Fig. 4a).

Cells within a tumor environment, in addition to experiencing higher oxidative stress, also show changes in efflux transporters. One family of efflux transporters whose upregulation has been linked to drug resistance are the ATP-cassette binding proteins (ABC transporters) [57]. PCR analysis revealed that the *ABC-B1* mRNA expression was elevated in STEMs and was almost 3-fold greater when compared to tri-cultures of A549/HPMEC/MSCs in 2-D (Fig. 4b). This finding prompted us to investigate the vulnerability of STEMs to dose escalation of two common chemotherapeutic agents, paclitaxel and gemcitabine. STEMs and 2-D tri-cultures were exposed to escalating doses of paclitaxel (1, 10, 100, and 1000 nM) and gemcitabine (1, 10, 30, and 100 μM), and the cell viability was assessed by MTT 48 h after continuous exposure (Fig. 4c and d). In the case of paclitaxel, at a low dose of 1 nM, no statistically significant difference in cell survival was observed between STEMs and control 2-D tri-cultures. However, at a dose of 10 nM a statistically significant (*p* = 0.0152), increase in cell survival was observed in the STEMs versus 2-D tri-cultures. Further dose escalation to 1000 nM did not result in any significant differences in cell survival in STEMs versus 2-D. In the case of gemcitabine, there were no statistically significant differences in cell survival until a dose of 30 μM. Above this dose, a consistently 10 % higher viability was observed in STEMs versus 2-D. This difference, although modest, was statistically significant with p value of 0.0039 for 100 μM. Nevertheless, the absence of much larger differences in cell survival in STEMs versus 2-D cultures is an unexpected finding in view of the elevated ROS levels and upregulation in *ABC-B1*. One possible explanation could be that MSCs confer some protective effect on the cells. However, another likely explanation is that multiple drug resistance (MDR) requires additional inputs, perhaps in the form of immunomodulation and soluble signals. This aspect of the STEM characteristic therefore, needs further refinement.

## Conclusions

Synthetic tumor microenvironment mimics, STEMs, composed of human lung epithelial, human lung endothelial, and human marrow-derived mesenchymal cells, were prepared using the hanging drop method, and characterized for their cellular and matrix characteristics (morphology, cell composition, and FN, CK18, vimentin, and CD31 expression), ROS production, and expression of drug resistance marker *ABC-B1*. After 15 days, STEMs showed many interesting characteristics including a hypoxic core that was devoid of epithelial cells but dominated by MSCs and a small but viable population of endothelial cells appear to be closely associated with MSCs in the hypoxic core. Immunohistochemistry revealed that, while FN expression was strong throughout, discrete regions that were positive for CK-18 and vimentin were present. Additionally, cells within STEMs showed high levels of ROS and upregulation of drug-resistance phenotype associated marker such as *ABC-B1*. In spite of the upregulation of marker associated with MDR, no appreciable differences in cell viability were observed between STEMs and 2-D tri-cultures in response to dose escalation of paclitaxel and gemcitabine. This unexpected finding suggests that the MDR phenotype might additionally require immune modulation and soluble signals. Nevertheless, the epithelial/endothelial/MSC 3-D culture model described herein bears many unique traits associated with cancer environments, and presents a useful platform for understanding tumor biology and drug screening.

## Additional files

Additional file 1: Figure S1. Immunohistochemical staining showing negative controls in 3-D and 2-D Triculture (Scale bar: 100 μm). (DOCX 343 kb) Additional file 2: Figure S2. Immunohistochemical staining against cytokeratin 18 in A549, human pulmonary microvascular endothelial cells (HPMEC), mesenchymal stem cells (MSCs) and 2-D Triculture (Scale bar: 100 μm for low magnification and 50 μm for high magnification images). (DOCX 1749 kb)
