# Supplementary material for: Recapitulating epithelial tumor microenvironment in vitro using three dimensional tri-culture of human epithelial, endothelial, and mesenchymal cells
Source: BMC Cancer. 2016 Aug 2;16:581. doi: 10.1186/s12885-016-2634-1 (PMC4971675; doi:10.1186/s12885-016-2634-1)
Supplement: Additional file 1: Figure S1. — Immunohistochemical staining showing negative controls in 3-D and 2-D Triculture (Scale bar: 100 μm). (DOCX 343 kb) [file 12885_2016_2634_MOESM1_ESM.docx]

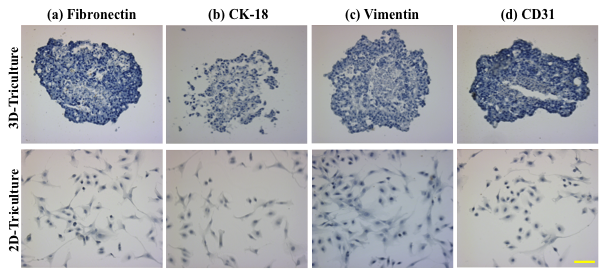


**Additional file 1: Figure S1:** Immunohistochemical staining showing negative controls in 3-D and 2-D Triculture (Scale bar: 100 µm).
