# Supplementary material for: Recapitulating epithelial tumor microenvironment in vitro using three dimensional tri-culture of human epithelial, endothelial, and mesenchymal cells
Source: BMC Cancer. 2016 Aug 2;16:581. doi: 10.1186/s12885-016-2634-1 (PMC4971675; doi:10.1186/s12885-016-2634-1)
Supplement: Additional file 2: Figure S2. — Immunohistochemical staining against cytokeratin 18 in A549, human pulmonary microvascular endothelial cells (HPMEC), mesenchymal stem cells (MSCs) and 2-D Triculture (Scale bar: 100 μm for low magnification and 50 μm for high magnification images). (DOCX 1749 kb) [file 12885_2016_2634_MOESM2_ESM.docx]

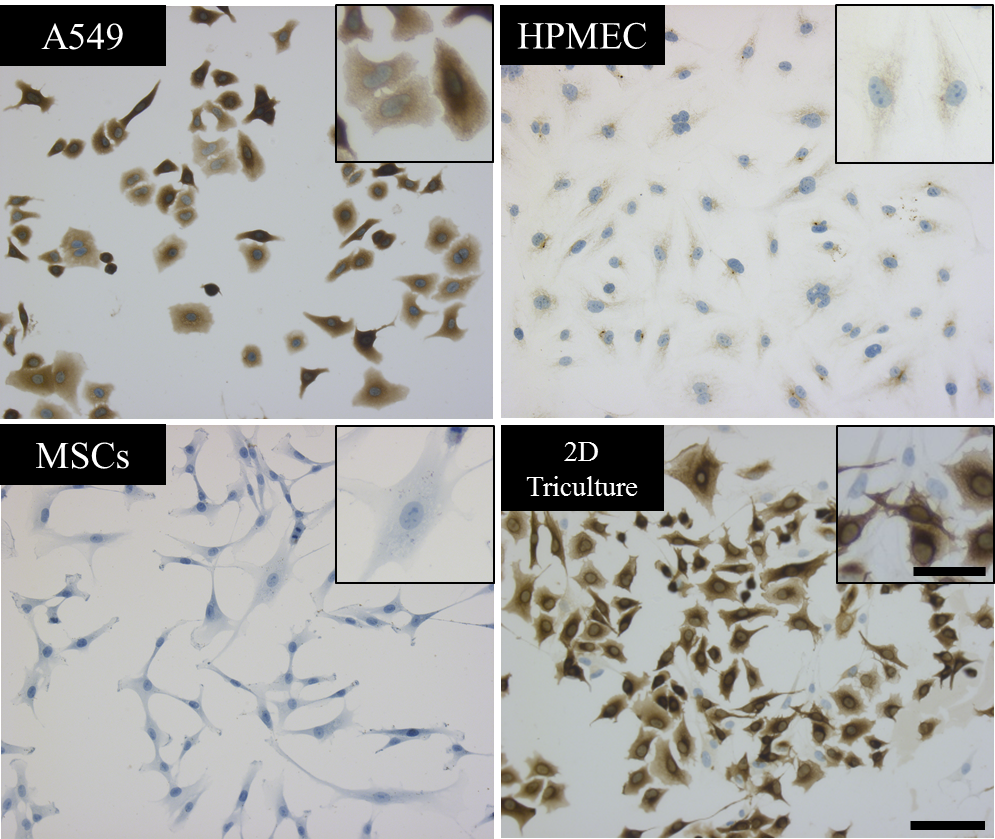


**Additional file 2: Figure S2:** Immunohistochemical staining against cytokeratin 18 in A549, human pulmonary microvascular endothelial cells (HPMEC), mesenchymal stem cells (MSCs) and 2-D Triculture (Scale bar: 100 µm for low magnification and 50 µm for high magnification images).
